# Protective Effects of *Lactobacillus plantarum* Lac16 on *Clostridium perfringens* Infection-Associated Injury in IPEC-J2 Cells

**DOI:** 10.3390/ijms222212388

**Published:** 2021-11-17

**Authors:** Yuanhao Zhou, Baikui Wang, Qi Wang, Li Tang, Peng Zou, Zihan Zeng, Huihua Zhang, Li Gong, Weifen Li

**Affiliations:** 1 Key Laboratory of Molecular Animal Nutrition of the Ministry of Education, Key Laboratory of Animal Nutrition and Feed Science (Eastern of China) of the Ministry of Agriculture, Key Laboratory of Animal Feed and Nutrition of Zhejiang Province, Institute of Animal Nutrition and Feed Sciences, College of Animal Sciences, Zhejiang University, Hangzhou 310058, China; zyh17767072477@163.com (Y.Z.); wangbaikui@zju.edu.cn (B.W.); 13430230952@163.com (Q.W.); 11717015@zju.edu.cn (L.T.); 21917085@zju.edu.cn (P.Z.); 21817016@zju.edu.cn (Z.Z.); 2 Department of Animal Sciences, School of Life Science and Engineering, Foshan University, Foshan 528225, China; hhzhang2@163.com

**Keywords:** *Lactobacillus plantarum*, *Clostridium perfringens*, cellular injury, intestinal barrier integrity, inflammation

## Abstract

*Clostridium perfringens* (*C. perfringens*) causes intestinal injury through overgrowth and the secretion of multiple toxins, leading to diarrhea and necrotic enteritis in animals, including pigs, chickens, and sheep. This study aimed to investigate the protective effects of *Lactobacillus plantarum* (*L. plantarum*) Lac16 on *C. perfringens* infection-associated injury in intestinal porcine epithelial cell line (IPEC-J2). The results showed that *L. plantarum* Lac16 significantly inhibited the growth of *C. perfringens*, which was accompanied by a decrease in pH levels. In addition, *L. plantarum* Lac16 significantly elevated the mRNA expression levels of host defense peptides (HDPs) in IPEC-J2 cells, decreased the adhesion of *C. perfringens* to IPEC-J2 cells, and attenuated *C. perfringens*-induced cellular cytotoxicity and intestinal barrier damage. Furthermore, *L. plantarum* Lac16 significantly suppressed *C. perfringens*-induced gene expressions of proinflammatory cytokines and pattern recognition receptors (PRRs) in IPEC-J2 cells. Moreover, *L. plantarum* Lac16 preincubation effectively inhibited the phosphorylation of p65 caused by *C. perfringens* infection. Collectively, probiotic *L. plantarum* Lac16 exerts protective effects against *C. perfringens* infection-associated injury in IPEC-J2 cells.

## 1. Introduction

*Clostridium perfringens* (*C. perfringens*) is a Gram-positive, spore-forming, anaerobic, rod-shaped bacterium [1,2], which can be isolated from the natural environment (e.g., soil) and intestines of human and animals as a component of the normal microbial community [3,4]. However, *C. perfringens* is an opportunistic pathogen that can cause various intestinal diseases under certain conditions, such as its overgrowth or disruption of the intestinal microbiota [5,6,7]. *C. perfringens* strains could secrete more than twenty toxins or enzymes, which are principal virulence factors [8]. Based on the secretion of four major toxins (α, β, ε, and ι), *C. perfringens* can be grouped into five different toxin types (types A to E) [9]. Among them, *C. perfringens* type A is very common in the intestines of warm-blooded animals; however, it also could cause intestinal diseases in domestic animals, such as pigs, chickens, and sheep [3,10]. Its symptoms in piglets include severe diarrhea, accompanied by necrotic mucosa and intestinal villi atrophy [11,12]. It is worth mentioning that antibiotics play a critical role in the prevention and clinical treatment of diseases caused by *C. perfringens* [13,14]. However, under the situation of feed antibiotics prohibition, *C. perfringens* infection has become an important problem, hindering the development of the pig industry [13,15,16]. Nowadays, many feed additives are used in animal husbandry to prevent *C. perfringens* infection, such as probiotics, prebiotics, symbiotics, essential oils, and herbs [17,18,19,20].

Probiotics are defined as “live microorganisms that, when administered in adequate amounts, confer a health benefit on the host” [21]. In animal husbandry, beneficial effects of probiotics are mainly reflected in nutrition and immunity [22]. They promote animal health by regulating immune functions and maintaining the dynamic balance of intestinal microbiota [23,24]. Some studies reported that probiotics, especially *Lactobacillus* spp., exerted protective effects against *C. perfringens* infection in vivo and in vitro. For example, *Lactobacillus acidophilus* (*L. acidophilus*) and *L. fermentum* inhibited the growth and α-toxin production capacity of *C. perfringens* in vitro [25]. Furthermore, *L. fermentum*, *L. salivarius*, *L. plantarum*, and *L. acidophilus* inhibited *C. perfringens* infection-associated necrotic enteritis of broilers by improving intestinal barrier integrity, reducing lesions, ameliorating inflammation, as well as modulating intestinal microbiota [26,27,28,29].

Our previous study also found that *L. plantarum* Lac16 effectively protected broilers against *C. perfringens* infection [30]. However, it has not been proved whether *L. plantarum* Lac16 can protect pig intestines in cases of *C. perfringens* infection. The intestinal porcine epithelial cell line (IPEC-J2) has exhibited a high specificity in pig studies and is a suitable model for investigating the interactions between bacteria and intestinal epithelia in vitro [31,32]. Thus, this study aimed to investigate the protective effects of *L. plantarum* Lac16 on *C. perfringens* infection-associated injury in IPEC-J2 cells.

## 2. Results

### 2.1. L. plantarum Lac16 Inhibited the Growth and Biofilm Formation of C. perfringens

**Figure 1:**
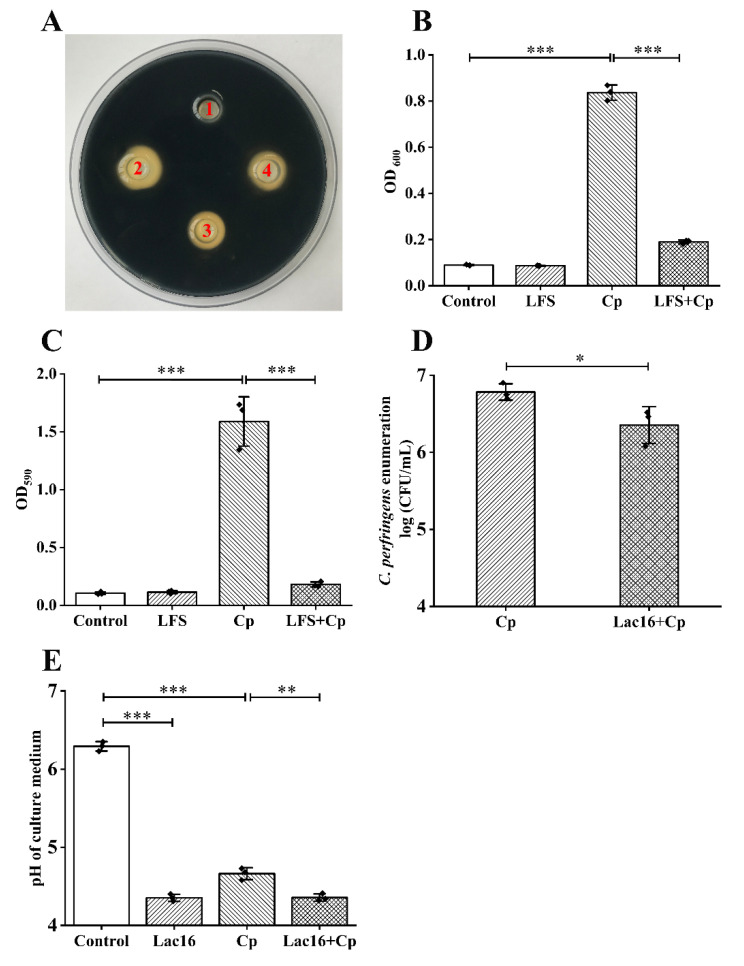
Antimicrobial activity of *L. plantarum* Lac16 on *C. perfringens*. (**A**) Agar well diffusion assay—1: de Man–Rogosa–Sharpe (MRS) broth; 2: 100 µg/mL of ampicillin; 3 and 4: LFS. (**B**) The growth of *C. perfringens* in different groups was measured at OD600 after 12 h of incubation. (**C**) Biofilm formation was measured at OD590. (**D**) *C. perfringens* levels in the co-culture experiment. (**E**) pH values of cultures in different groups. Data are presented as the means ± SD for *n* = 3; * *p* < 0.05, ** *p* < 0.01, *** *p* < 0.001 (*t* test).

Probiotics could produce antimicrobial substances and lower the environmental pH to resist pathogen infections [25]. As shown in Figure 1A, the positive group (100 µg/mL of ampicillin) exhibited the best bacteriostatic effect on *C. perfringens*, while *L. plantarum* Lac16 fermentation supernatant (LFS) also exhibited good bacteriostatic effects, forming a clear boundary of bacteriostatic zone. At the same time, as shown in Figure 1B, after 12 h of culture, the optical density (OD_600_) of the CP group increased sharply. However, in the group of *C. perfringens* co-incubated with the LFS, the OD_600_ was significantly lower than that of the CP group (*p* < 0.001), indicating that LFS could inhibit the proliferation of *C. perfringens*. In addition, LFS significantly inhibited the biofilm formation of *C. perfringens* (*p* < 0.001; Figure 1C). At the same time, we found that the inhibitory effects of *L. plantarum* Lac16 are different from those of some other probiotics (e.g., *Bacillus licheniformis* (*B. licheniformis*), *B. coagulans*, *C. butyricum*) that could not inhibit the growth (*p* > 0.05; Figures S1 and S2A) and biofilm formation of *C. perfringens* (*p* > 0.05; Figure S2B). Moreover, the colony forming units (CFUs) of *C. perfringens* in the co-culture group were significantly decreased when compared to the CP group (*p* < 0.05; Figure 1D), and the pH value in the co-culture group was lower than that of the CP group (*p* < 0.01; Figure 1E).

### 2.2. L. plantarum Lac16 Enhanced mRNA Expression of Host Defense Peptides (HDPs) in IPEC-J2 Cells

**Figure 2:**
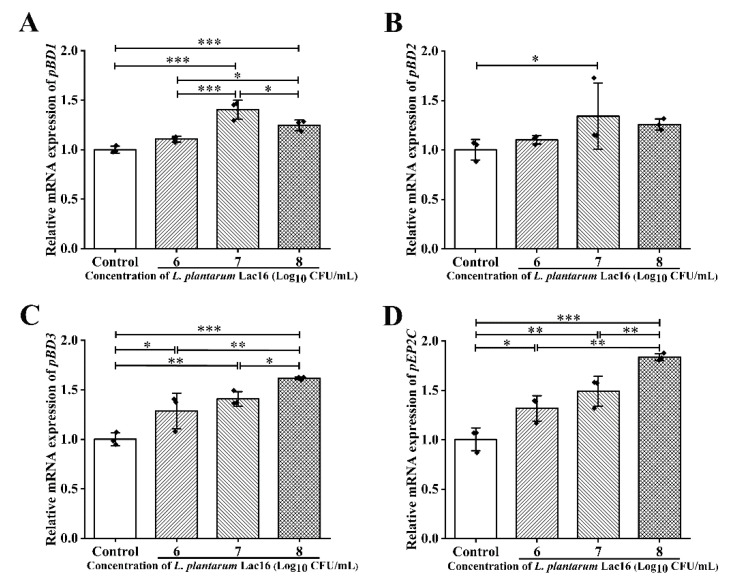
Gene expression levels of HDPs in *L. plantarum* Lac16-treated IPEC-J2 cells. (**A**) *pBD1*, (**B**) *pBD2*, (**C**) *pBD3*, (**D**) *pEP2C*. mRNA expression was standardized to *β-actin* expression. Data are presented as the means ± SD for *n* = 3; * *p* < 0.05, ** *p* < 0.01, *** *p* < 0.001 (one-way ANOVA).

It is well known that intestinal epithelial cells and phagocytes could secrete HDPs to maintain the homeostasis in the intestine [33,34,35]. Co-incubation with *L. plantarum* Lac16 could increase the expression levels of HDP genes in IPEC-J2 cells, including porcine β-defensin (*pBD1*, *pBD2*, *pBD3)* and porcine epididymis protein 2 splicing variant C (*pEP2C*) (Figure 2). Specifically, the highest expression level of *pBD1* was observed in the group co-incubated with *L. plantarum* Lac16 at the concentration of 10^7^ CFU/mL (*p* < 0.001; Figure 2A). At the same time, the trend in *pBD2* gene expression level was similar (*p* < 0.05, Figure 2B). Gene expression levels of *pBD3* and *pEP2C* were significantly elevated in a concentration-dependent manner of *L. plantarum* Lac16 (*p* < 0.05; Figure 2C,D). Therefore, the concentration (10^7^ CFU/mL) of *L. plantarum* Lac16 was selected for the follow-up experiments.

### 2.3. L. plantarum Lac16 Alleviated C. perfringens Infection-Associated Lactate Dehydrogenase (LDH) Leakage

**Figure 3:**
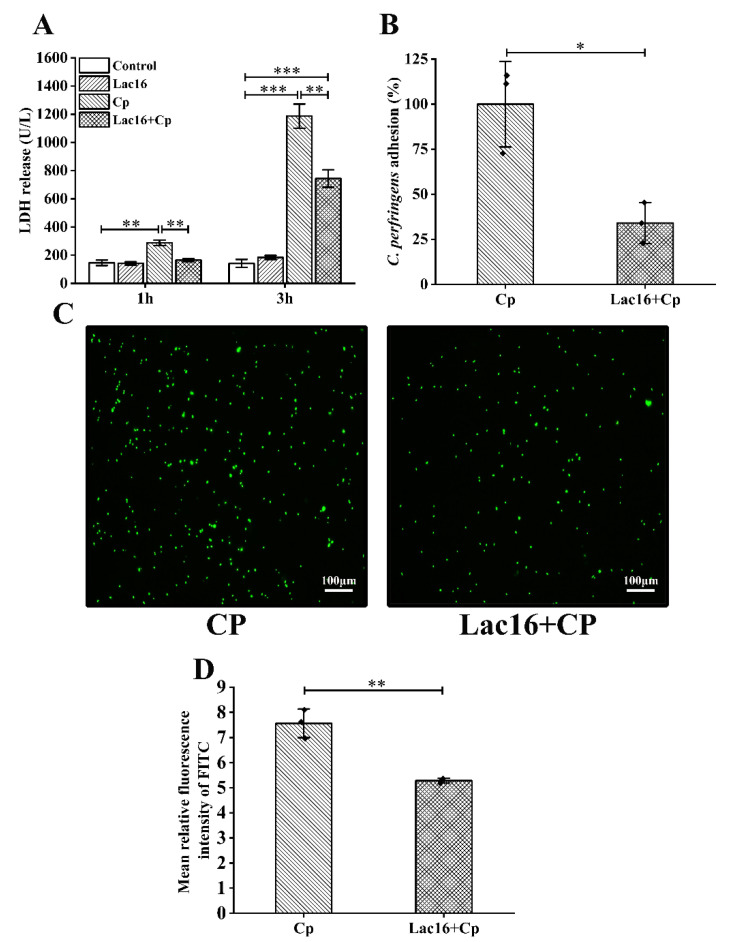
Cytotoxicity and *C. perfringens* adhesion assays. (**A**) Concentrations of LDH in the supernatants of IPEC-J2 cells. (**B**) Adhesions of *C. perfringens* to IPEC-J2 cells were detected by tryptose sulfite cycloserine (TSC) agar. Adherence ratio of the *C. perfringens* group was normalized to 100%. (**C**) Adhesions of *C. perfringens* (green) to IPEC-J2 cells were detected by fluorescence labeling method. (**D**) The mean relative fluorescence intensity of fluorescein isothiocyanate (FITC) in panel C. Data are presented as the means ± SD for *n* = 3; * *p* < 0.05, ** *p* < 0.01, *** *p* < 0.001 (*t* test).

LDH leakage is an important indicator of cell membrane damage [36]. When the intestinal epithelial cells are infected with *C. perfringens*, LDH, which is a stable cytoplasmic enzyme, will rapidly release into the extracellular environment [25,37]. The results of LDH leakage showed that pretreatment with *L. plantarum* Lac16 did not increase LDH release (*p* > 0.05; Figure 3A), whereas LDH release in the CP group was significantly elevated (*p* < 0.01), which was alleviated by preincubation with *L. plantarum* Lac16 (*p* < 0.01). At the same time, it is worth mentioning that *C. butyricum*, a probiotic which had no inhibitory effect on *C. perfringens* in our previous study, also did not possess this alleviated effect on the increased LDH release (*p* > 0.05; Figure S2C). In addition, the increased LDH release induced by *C. perfringens* infection for 3 h was not alleviated to normal levels by *L. plantarum* Lac16 preincubation (*p* < 0.001). Therefore, for subsequent experiments, IPEC-J2 cells infected with *C. perfringens* for 1 h were selected as the time point for sampling.

### 2.4. L. plantarum Lac16 Suppressed the Adhesion of C. perfringens to IPEC-J2 Cells

In the occurrence of infection, pathogens need to adhere to the host epithelial cells [38]. For the bacterial adhesion assay, the adhesion rate in the CP group (IPEC-J2 cells were only co-incubated with *C. perfringens*) was defined as 100% (Figure 3B). However, preincubation with *L. plantarum* Lac16 could significantly decrease the adhesion rate of *C. perfringens* to IPEC-J2 (*p* < 0.05). Similar results were obtained in the images of the fluorescence labeling method (Figure 3C). The quantity of fluorescent labeled pathogens that adhered to IPEC-J2 cells in the CP group was significantly larger than that of the group pretreated with *L. plantarum* Lac16 (*p* < 0.01; Figure 3D).

### 2.5. L. plantarum Lac16 Attenuated C. perfringens-Induced Damage to Intestinal Epithelial Barrier Function

**Figure 4:**
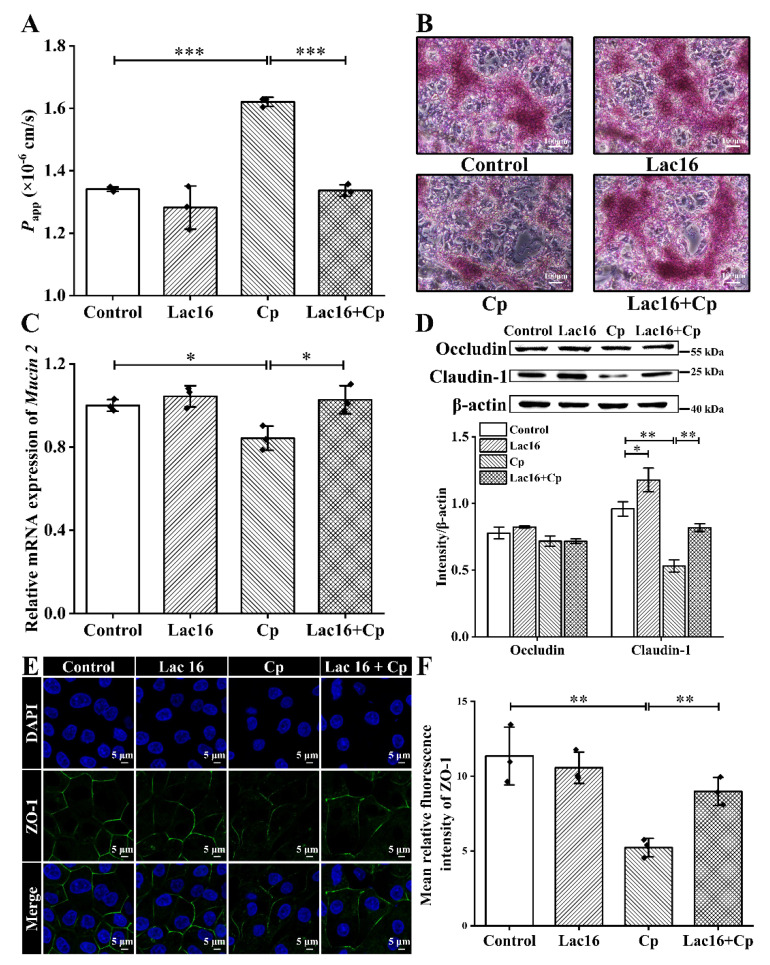
Intestinal barrier functions and tight junction proteins of IPEC-J2 cell monolayers. (**A**) *P*_app_ of IPEC-J2 monolayers based on apical-to-basolateral flux of fluorescein sodium. (**B**) Mucin production was detected by PAS staining (Mucin, carmine). (**C**) Relative gene expression level of *Mucin 2*. mRNA expression was standardized to *β-actin* expression. (**D**) Western blot detection of occludin and claudin-1 in IPEC-J2 cells, and the quantitative analysis of the expression levels of bands. *β*-actin was used as an indicator of protein loading. (**E**) Immunofluorescence staining of zona occludens-1 (ZO-1, green) in IPEC-J2 cells. Nuclei were counterstained using 4′,6-Diamidino-2-phenylindole dihydrochloride (DAPI, blue). All images were obtained at 63× magnification in oil. (**F**) The mean relative fluorescence intensity of ZO-1 in panel E. Data are presented as the means ± SD for *n* = 3; * *p* < 0.05, ** *p* < 0.01, *** *p* < 0.001 (*t* test).

To evaluate *C. perfringens*-induced damage to intestinal epithelial barrier function and the corresponding protective effects of *L. plantarum* Lac16, we determined the flux of fluorescein sodium, mucin production, and the expression levels of tight junction proteins in IPEC-J2 cells. When cells developed tight junctions and completely differentiated into tight monolayers, they were infected with *C. perfringens*. The significant increased flux of fluorescein sodium was observed in the CP group (*p* < 0.001; Figure 4A). It is worth mentioning that fluorescein sodium is not transported through the tight monolayers by membrane transporters or channels expressed in the mammalian epitheliums, but leaks through the intercellular cracks formed after tight junction injury [39,40]. However, *L. plantarum* Lac16 pretreatment significantly alleviated the increase in apparent permeability coefficient (*P*app) of the IPEC-J2 monolayers induced by *C. perfringens* infection (*p* < 0.001). In addition, the results of periodic acid–Schiff (PAS) staining showed that *C. perfringens* infection significantly inhibited mucin production, which was effectively reversed by *L. plantarum* Lac16 pretreatment (Figure 4B). At the same time, *C. perfringens* infection sharply decreased the mRNA expression level of *Mucin 2* in IPEC-J2 cells (*p* < 0.05), while it was effectively alleviated by preincubation with *L. plantarum* Lac16 (*p* < 0.05; Figure 4C).

The results of the expression levels of tight junction proteins in IPEC-J2 cells showed that, compared with the control group, the expression of claudin-1 was significantly elevated by *L. plantarum* Lac16 treatment (*p* < 0.05), whereas it was significantly suppressed by *C. perfringens* infection (*p* < 0.01; Figure 4D and Figure S3). At the same time, *L. plantarum* Lac16 preincubation significantly alleviated the decrease in protein expressions caused by *C. perfringens* infection (*p* < 0.01). However, there was no significant difference in occludin expression among all groups (*p* > 0.05). Regarding immunofluorescence images of ZO-1 (Figure 4E), it was found that ZO-1 expression level was not affected by *L. plantarum* Lac16, whereas it was significantly suppressed by *C. perfringens* infection (*p* < 0.01; Figure 4F). However, this phenomenon was attenuated by pretreatment with *L. plantarum* Lac16 (*p* < 0.01).

### 2.6. L. plantarum Lac16 Alleviated C. perfringens-Induced Inflammatory Response

**Figure 5:**
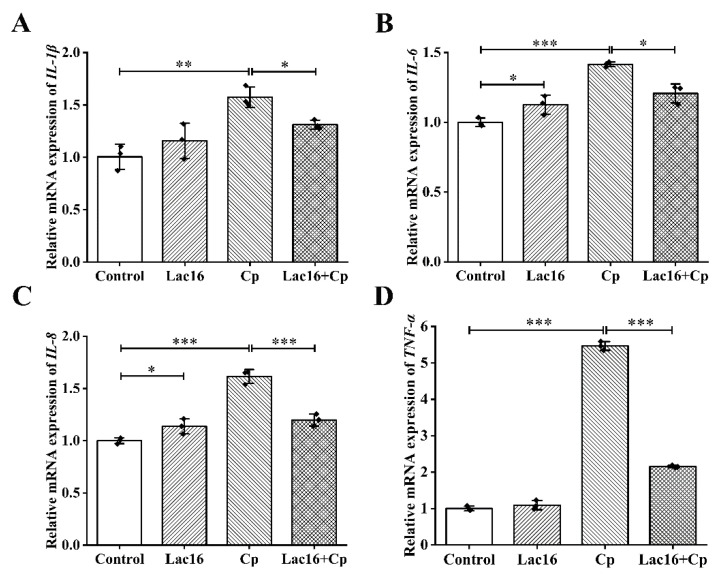
Relative gene expression levels of inflammatory cytokines during *C. perfringens* infection in IPEC-J2 cells preincubated with *L. plantarum* Lac16. (**A**) *IL-1β*, (**B**) *IL-6*, (**C**) *IL-8*, (**D**)*TNF-α*. mRNA expression was standardized to *β-actin* expression. Data are presented as the means ± SD for *n* = 3; * *p* < 0.05, ** *p* < 0.01, *** *p* < 0.001 (*t* test).

One of the salient features of pathogen infections in the host immune system is the intense inflammatory response [41,42]. The results of mRNA expression levels of inflammatory cytokines in IPEC-J2 cells showed that *L. plantarum* Lac16 could significantly elevate mRNA expression levels of *interleukin* (*IL*)-*6* and *IL-8* (*p* < 0.05; Figure 5B,C), while *C. perfringens* infection sharply elevated gene expression levels of *IL-1β*, *IL-6*, *IL-8*, and *tumor necrosis factor* (*TNF*)-*α* much more (*p* < 0.01; Figure 5A–D), which was significantly alleviated by preincubation with *L. plantarum* Lac16 (*p* < 0.05), especially for *IL-8* and *TNF-α* (*p* < 0.001).

### 2.7. L. plantarum Lac16 Alleviated the Increase in mRNA Expression Levels of Pattern Recognition Receptors (PRRs) Induced by C. perfringens Infection

**Figure 6:**
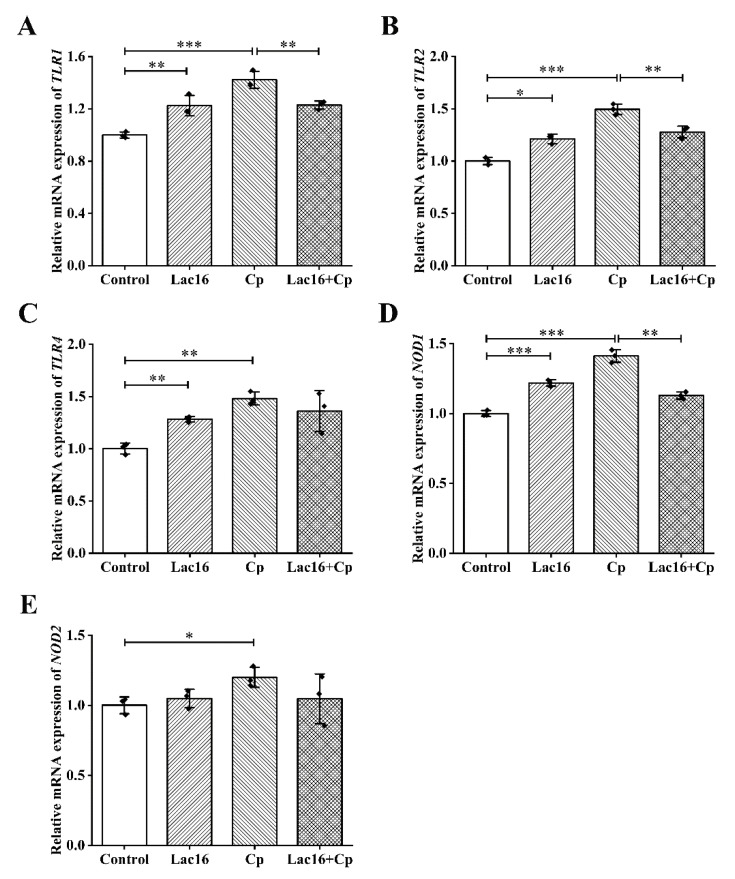
Relative gene expression levels of PRRs during *C. perfringens* infection in IPEC-J2 cells preincubated with *L. plantarum* Lac16. (**A**) *TLR1*, (**B**) *TLR2*, (**C**) *TLR4*, (**D**) *NOD1*, (**E**) *NOD2*. mRNA expression was standardized to *β-actin* expression. Data are presented as the means ± SD for *n* = 3; * *p* < 0.05, ** *p* < 0.01, *** *p* < 0.001 (*t* test).

Intestinal epithelial cells could express PRRs, which enable them to act as dynamic sensors of the microbial environment and foreign antigens [43]. PRRs, including Toll-like receptors (TLRs) and NOD-like receptors (NLRs), could initiate the activation of intracellular signaling pathways, thereby increasing the expression and release of chemokines, cytokines, and antibacterial peptides [44]. The results of mRNA expression levels of PRRs in IPEC-J2 cells showed that both *L. plantarum* Lac16 and *C. perfringens* could significantly up-regulate mRNA expression levels of TLRs (*p* < 0.05; Figure 6A–C). The most significant increase in mRNA expression levels of TLRs was in the group infected with *C. perfringens* only. However, the increased mRNA expression (*TLR1* and *TLR2*) caused by *C. perfringens* infection was effectively inhibited (*p* < 0.01) by co-incubation with *L. plantarum* Lac16. The trend in mRNA expression levels of nucleotide-binding oligomerization domains (NODs) was similar to that of TLRs (Figure 6D,E). Although both bacteria increased *NOD1* gene expression (*p* < 0.001), preincubation with *L. plantarum* Lac16 significantly reduced the increased gene expression associated with *C. perfringens* infection (*p* < 0.01; Figure 6D). However, this effect was not significant in *NOD2* gene expression (*p* > 0.05; Figure 6E).

### 2.8. L. plantarum Lac16 Alleviated Inflammatory Response Induced by C. perfringens Infection by Nuclear Factor Kappa B (NF-κB) Signaling Pathways

**Figure 7:**
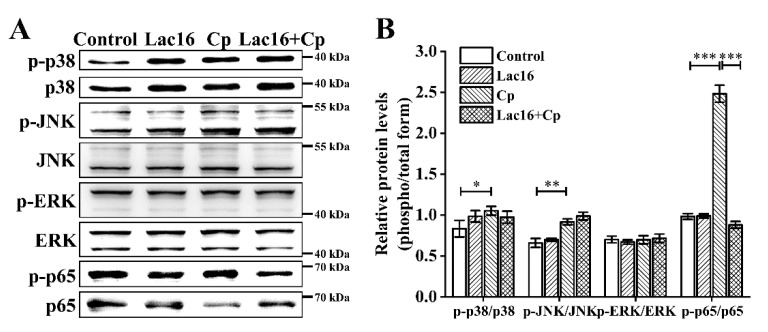
Western blot detection of MAPK and NF-κB pathways in IPEC-J2 cells. (**A**) Western blot analysis of MAPK and NF-κB pathways. (**B**) Quantitative analysis of the expression levels of phospho-p38/p38, phospho-JNK/JNK, phospho-ERK/ERK, and phosphor-p65/p65. Data are presented as the means ± SD for *n* = 3; * *p* < 0.05, ** *p* < 0.01, *** *p* < 0.001 (*t* test).

To investigate the potential signaling pathway that led to the release of inflammatory cytokines, phosphorylation levels of certain proteins of the mitogen-activated protein kinase (MAPK) and NF-κB were determined (Figure 7 and Figure S3). It was found that *C. perfringens* infection enhanced the phosphorylation of p38, c-Jun NH 2-terminal kinase (JNK), as well as p65 when compared to the control group (*p* < 0.05). Moreover, preincubation with *L. plantarum* Lac16 significantly inhibited *C. perfringens* infection-associated p65 phosphorylation (*p* < 0.001). In addition, the phosphorylation of extracellular signal-regulated kinase (ERK) in each group did not change significantly (*p* > 0.05).

## 3. Discussion

Probiotics provide benefits to the host through various mechanisms, including producing anti-bacterial substances, competing with pathogenic microorganisms for enterocyte binding, regulating the secretion of pro- and anti-inflammatory cytokines, and maintaining intestinal barrier integrity [45]. Several studies indicated that *Lactobacillus* spp. could prevent the infection of pathogenic bacteria, including *Escherichia coli*, *C. perfringens*, and *Salmonella Enteritidis* [25,45,46,47]. Previously, we found that *L. plantarum* Lac16 exhibited protective effects against *C. perfringens* infection in broilers [30]. In this study, we have shown that *L. plantarum* Lac16 inhibits the growth of *C. perfringens* and attenuates *C. perfringens* infection-associated intestinal injury in IPEC-J2 cells.

Probiotics exert antimicrobial activities by secreting antimicrobial substances, such as bacteriocins, organic acids, and hydrogen peroxide [48]. Cell-free supernatants contain antimicrobial substances secreted by probiotics, which can effectively inhibit the growth of various pathogens [49]. In addition, *C. perfringens* strains form biofilms to enhance their persistence and increase resistance to various stressors, such as oxidative and antibiotic stress [50], and this phenomenon has been proved to be involved in a large proportion of bacterial infections [51]. In the current study, it was found that the fermentation supernatant of *L. plantarum* Lac16 could inhibit the growth of *C. perfringens*, and significantly suppress *C. perfringens* biofilm formation. Moreover, the antimicrobial activity of *L. plantarum* is associated with the production of organic acids, which decreases environmental pH [52]. *C. perfringens* is a pH sensitive bacterium [53]. Acidic environments down-regulate the expression of virulence factors of *C. perfringens* while inhibiting its growth [53,54]. These findings indicate that *L. plantarum* Lac16 and its metabolites decreased environmental pH and inhibited biofilm formation as well as the growth of *C. perfringens*.

HDPs, as important components of the innate immune system, play critical roles in infection resistance [33]. They are mainly secreted by intestinal epithelial cells and phagocytes in the gastrointestinal tract [34]. HDPs are involved in the maintenance of intestinal homeostasis and innate immune defenses during infection through multiple mechanisms. Specifically, HDPs secreted by intestinal epithelial cells exert direct antimicrobial effects on invading bacterial pathogens and intestinal microbiota [35]. Several studies indicated that *Lactobacillus* spp. enhance the expression of HDPs [55,56,57]. Increased secretion of endogenous HDPs improves early immune system responses to pathogenic infections and inflammation [58]. In this study, *L. plantarum* Lac16 promoted the expression of endogenous HDPs in IPEC-J2 cells, which is consistent with results observed by Wang et al. that *L. plantarum* ZLP001 enhanced intestinal defense responses by promoting the secretion of HDPs [34].

LDH is a stable cytoplasmic enzyme that possesses oxidation–reduction activities. When cells are subjected to cell membrane damage that is caused by intracellular or extracellular stress, LDH will rapidly release into the extracellular environment [37]. Alpha toxin, which is produced by *C. perfringens* type A, can result in extensive degradation of the plasma membrane, leading to LDH release [59]. Elevated LDH release is a key feature of apoptosis, necrosis, and other forms of cellular damage [60,61]. In this study, it was found that *C. perfringens* infection elevated LDH release from IPEC-J2 cells, implying that *C. perfringens* damaged the intestinal epithelial cells, resulting in intracellular enzyme leakage. At the same time, when cells were infected with *C. perfringens* for 1 h, the increased LDH release was effectively alleviated to the normal level by *L. plantarum* Lac16 preincubation. This phenomenon may be related to *L. plantarum* Lac16 directly inhibiting the growth of *C. perfringens* and suppressing *C. perfringens* adhesion to IPEC-J2 cells, and indirectly inducing IPEC-J2 cells to produce antimicrobial substances to resist *C. perfringens* infection, such as HDPs. Similar protective results have been reported by some studies [62,63,64,65]. However, when the cells were infected with *C. perfringens* for 3 h, *L. plantarum* Lac16 preincubation did not reduce the LDH release to the normal level, implying that the damage of *C. perfringens* to intestinal epithelial cells was beyond the protective effects of *L. plantarum* Lac16 preincubation.

Pathogenic adherence to host epithelial cells is an indispensable step in the occurrence of infection [38]. In the meantime, *Lactobacillus* could effectively prevent pathogenic adhesion to intestinal epithelial cells and play an important role in maintaining intestinal homeostasis [66]. Thus, one way for *Lactobacillus* to exert its antibacterial activity is by occupying the adhesion site of pathogens to intestinal epithelial cells [67]. Probiotic adhesion to intestinal epithelial cells can optimize the balance and activities of intestinal microbiota [68]. It is worth mentioning that the adherence of *C. perfringens* strains increases toxin production [69]. In this study, *L. plantarum* Lac16 significantly suppressed *C. perfringens* adhesion to IPEC-J2 cells, thereby resisting *C. perfringens* infection and protecting intestinal epithelial cells. This finding is similar to that of another study, which showed that *L. rhamnosus* effectively inhibited the adhesion of *C. perfringens* to pig intestinal mucosa [70].

Epithelial cells form a layer that acts as a physical barrier connected by tight junctions between each cell [71]. In addition, the mucus layer covering the surface of the intestinal epithelium also plays a crucial role in protecting intestinal epithelial barrier integrity [72]. The main components of tight junction proteins are claudins, zona occludens, and occludin [73]. Tight junctions regulate the paracellular transport of various substances, such as ions, solutes, molecules, and water, across the intestinal epithelium [74], thereby maintaining physiological functions of epithelial cells [75]. Probiotics and pathogens can alter the expression of tight junctions [75,76]. Moreover, the dysregulation of tight junction protein integrity enhances intestinal barrier permeability [77]. It is worth noting that virulence factors, which are produced by *C. perfringens*, could effectively impair tight junctions [15,78]. In this study, *L. plantarum* Lac16 preincubation attenuated *C. perfringens*-induced disruption of mucus production. In addition, *C. perfringens* infection suppressed the expression levels of claudin-1 and ZO-1, which were effectively alleviated by *L. plantarum* Lac16 preincubation. Our results are consistent with those of studies reporting on the effects of probiotics in the alleviation of intestinal barrier dysfunction caused by pathogens [45,72,79]. It is worth mentioning that *C. perfringens* enterotoxin (CPE), a pore-forming toxin that could disrupt the selective permeability of the plasma membrane of target cells and result in cell death, interacts with claudins to impair tight junction barrier function [80]. At the same time, scaffolding proteins such as ZO-1 and signaling proteins are associated with tight junctions by binding their PDZ-domains to respective binding motifs at the C-terminus of claudins [81,82]. In our opinion, the structure between ZO-1 and claudins causes them to have the same trend of change. However, we cannot give a reasonable explanation at present for the result that the protein expressions of occludin did not change. Moreover, it had been reported that it is not possible to investigate porcine intestinal barrier function well using the existing IPEC-J2 model because of its atypically high transepithelial resistances and low active transport rates [83]. These existing defects require us to find more suitable in vitro models or carry out animal experiments in future studies.

Infections with pathogenic microbes, such as *C. perfringens*, often lead to significant inflammatory responses [41,42]. Pro-inflammatory cytokines mediate inflammatory responses to invading pathogens through multiple modulatory mechanisms, such as lymphocyte activation, neutrophil migration, and cell proliferation [84]. However, excess secretion of pro-inflammatory cytokines has deleterious effects on the host [85]. In this study, although *L. plantarum* Lac16 elevated the expression levels of pro-inflammatory cytokines, such as *IL-6* and *IL-8*, preincubation with *L. plantarum* Lac16 significantly inhibited *C. perfringens*-associated inflammatory responses, which was consistent with our previous results that *L. plantarum* Lac16 alleviated *C. perfringens* infection-associated inflammatory responses in the ileum mucosa of broilers [30]. In general, we postulate that one of the mechanisms through which *L. plantarum* Lac16 protects intestinal epithelial cells from *C. perfringens* injury is by relieving inflammation.

PRRs, including TLRs and NLRs, are important receptor molecules in the host immune system [86]. PRRs play a crucial protective role in the immune system via identifying pathogen-associated molecular patterns (PAMPs), such as bacterial nucleic acids and flagellin, to resist pathogenic infections [87]. Furthermore, PRRs initiate the activation of intracellular signaling pathways, thereby increasing the expression and release of chemokines, cytokines, and antibacterial peptides [44]. It is worth mentioning that TLRs play a crucial role in the regulation of mucosal immune responses and the maintenance of intestinal homeostasis [88]. At the same time, when pathogens gain entry into the cytoplasm, NLRs have been shown to initiate innate immune responses [89]. In the current study, we found that *L. plantarum* Lac16 elevated the expression levels of TLRs. Changes in expression levels of TLRs in intestinal epithelial cells regulate *β*-defensin expression [90], corresponding to our above findings that *L. plantarum* Lac16 enhances the expression of endogenous HDPs. The *C. perfringens* challenge sharply elevated the mRNA expression levels of TLR1, TLR2, and TLR4, whereas preincubation with *L. plantarum* Lac16 attenuated this dramatic increase. Furthermore, *L. plantarum* Lac16 alleviated *C. perfringens*-induced elevations in NOD1 expression, in tandem with other studies showing that probiotics attenuate pathogen-associated elevations in PRR expression [45,91]. In our opinion, *L. plantarum* Lac16 activates PRR-dependent signaling pathways and strengthens the immune system to resist *C. perfringens* infection.

High expression levels of PRRs and cytokines are accompanied by the activation of MAPK and NF-κB signaling pathways [92,93]. MAPK signaling pathways are signal transduction modules that transform extracellular signals into intracellular responses that regulate the processes of cell growth, differentiation, and migration [94,95]. In addition, MAPK plays a crucial role in modulating the synthesis and release of inflammatory mediators during inflammatory responses [96,97]. Elevated expressions of PRRs enhance the phosphorylation of MAPK [98,99]. In addition, NF-κB, an important transcription factor, is a key factor for modulating the expression of genes and proteins involved in inflammatory responses [100]. For example, the production of proinflammatory cytokines, such as TNF-α, is closely associated with the activation of NF-κB [101]. Probiotics have also been shown to exert their protective mechanisms against pathogenic infections by modulating the NF-κB signaling pathways [45,102,103]. In the current study, *C. perfringens* infection significantly elevated p38, JNK, and p65 phosphorylation in IPEC-J2 cells. However, preincubation with *L. plantarum* Lac16 did not significantly attenuate *C. perfringens*-induced p38 and JNK phosphorylation, but significantly attenuated the increase in p65 phosphorylation. The above results indicated that *C. perfringens*-induced inflammatory responses are mediated by MAPK and NF-κB signaling pathways, and *L. plantarum* Lac16 prevents *C. perfringens* infection-associated excess immune responses by attenuating p65 NF-κB phosphorylation.

In summary, *L. plantarum* Lac16 attenuated *C. perfringens*-induced injury in IPEC-J2 cells via several mechanisms, including inhibiting the growth and biofilm formation of *C. perfringens*, preventing its adhesion to epithelial cells, promoting the expressions of endogenous host defense peptides, protecting intestinal epithelial barrier integrity, and alleviating inflammatory responses by attenuating p65 NF-κB phosphorylation. These findings highlight the significance of *L. plantarum* Lac16 as a potential therapeutic strategy against *C. perfringens* infection and provide a theoretical basis for the application of *L. plantarum* Lac16 in animal husbandry to resist pathogen infections.

## 4. Materials and Methods

### 4.1. Bacterial Strains and Culture Conditions

*L. plantarum* Lac16 was isolated by our laboratory and preserved at the China Center for Type Culture Collection (CCTCC, No. M2016259). *L. plantarum* Lac16 was cultured in MRS medium and incubated at 37 °C for 18 h. *C. perfringens* type A (ATCC 13124) was cultured in Reinforced clostridium medium (RCM; Hopebio, Qingdao, China) and incubated at 37 °C under anaerobic conditions for 18 h. The overnight-incubated bacterial cultures were centrifuged at 5000 rpm for 5 min. After being washed three times using sterile phosphate-buffered saline (PBS, pH = 7.2), bacteria were resuspended in PBS and their concentrations were determined using a standard curve. Then, they were diluted to a certain concentration and stored at 4 °C for further use.

### 4.2. Bacteriostasis Detection of LFS to C. perfringens

Agar diffusion was performed as previously described by Wang et al. [34] with some modifications to perform the bacteriostasis detection. Briefly, *L. plantarum* culture was centrifuged at 5000 rpm for 10 min to obtain the supernatant, which was then filtered through a 0.22 μm membrane to remove suspended bacteria and stored at 4 °C. About 0.2% (*v*/*v*) of the overnight culture of *C. perfringens* was added to TSC agar (Hopebio, Qingdao, China), which cooled down to about 50 °C. Then, the medium was well mixed and poured into plastic plates, which placed oxford cups in advance. The oxford cups were removed after the medium had solidified. Then, 100 µL of LFS was injected into each well, and plates were placed in anaerobic gas generating packs (Hopebio, Qingdao, China) for 12 h. This bacteriostatic experiment was performed in triplicate.

### 4.3. Determination of Biofilm Formation of C. perfringens

Biofilm formation of *C. perfringens* was determined as previously described by Jiang et al. [104] with some modifications. *L. plantarum* and *C. perfringens* were cultured in modified RCM medium (glucose content was increased to 20 g/L on the original basis) and incubated at 37 °C for 18 h, respectively. Then, the concentration of the *C. perfringens* culture was adjusted to 10^7^ CFU/mL using the medium, and the supernatant of *L. plantarum* was collected as described above. Experimental groups were treated as: Control: Sterile modified RCM medium (200 μL) was inoculated into 96-well culture plates; LFS: LFS (100 μL) and 100 μL of medium were inoculated; Cp: Resuspended *C. perfringens* (100 μL) and 100 μL of medium were inoculated; LFS + Cp: LFS (100 μL) and 100 μL of resuspended *C. perfringens* were inoculated, respectively. The 96-well culture plate was incubated in an anaerobic environment at 37 °C for 12 h, and the bacterial proliferation index was measured at OD_600_ using SpectraMax M5 (Molecular Devices, Sunnyvale, CA, USA). Then, bacterial cultures were removed with caution, and the bacteria in wells were gently washed thrice using PBS and incubated with 100 μL of 1% crystal violet for 30 min. After that, crystal violet was removed and wells were gently washed thrice. Then, 100 μL of 95% alcohol was added into the wells to dissolve excess crystal violet. At last, OD_590_ values in each well were measured. The higher the optical density, the more biofilm formation. Experiments were performed in triplicate.

### 4.4. Co-Culture Experiment and pH Determination of Bacterial Cultures

Bacterial co-culture experiments were performed as previously described by Guo et al. [25] with some modifications. *L. plantarum* or *C. perfringens* suspensions (100 μL, 10^7^ CFU/mL) or both 100 μL of them were inoculated in modified RCM medium (the final volume is 10 mL), respectively. The above cultures were incubated at 37 °C for 12 h, and their pH values were determined. At the same time, the cultures were serially diluted, cultured on TSC agar, and incubated at 37 °C for 12 h to enumerate CFU. These experiments were performed in triplicate.

### 4.5. IPEC-J2 Cells Culture

IPEC-J2 cells were cultured in Dulbecco’s modified Eagle’s F12 ham medium (DMEM/F12; Gibco, Waltham, MA, USA) supplemented with 10% (*v*/*v*) fetal bovine serum (FBS; Gibco, MA, USA), 100 μg/mL streptomycin, and 100 U/mL penicillin (Sigma-Aldrich, St Louis, MO, USA). Incubation was performed at 37 °C in an atmosphere of 90% humidity and 5% CO_2_. When cell confluence reached 80%, cells were digested using 0.25% trypsin–EDTA solution (Gibco, MA, USA) and seeded in cell culture plates.

### 4.6. Determination of Expression Levels of HDPs by Real-Time PCR

IPEC-J2 cells were seeded in 12-well cell culture plates (Corning Life Science, Acton, MA, USA) at a density of 5 × 10^5^ cells/well. *L. plantarum* cultures were centrifuged and resuspended in DMEM/F12 supplemented with 10% FBS and stored at 4 °C. When IPEC-J2 cells reached 80% confluence, they were co-incubated in cell culture media containing different concentrations of *L. plantarum* Lac16 (10^6^, 10^7^, and 10^8^ CFU/mL) for 6 h. After being washed three times using PBS, IPEC-J2 cells were lysed by RNAiso Plus (Takara, Dalian, China) to extract RNA. Expression levels of *pBD*1, *pBD2*, *pBD3*, and *pEP2C* were then determined by real-time PCR. At the same time, untreated IPEC-J2 cells were used as the control group. *β*-actin was selected as an endogenous control and relative gene expressions were analyzed using the 2^−ΔΔCt^ method [105]. All experiments were performed in triplicate.

### 4.7. Cytotoxicity Assay

IPEC-J2 cells were seeded in 12-well culture plates at a density of 5 × 10^5^ cells/well. When cells reached 80% confluence, they were incubated with or without *L. plantarum* (10^7^ CFU/mL) for 6 h, respectively. After being washed three times using PBS, cells were infected with *C. perfringens* (10^6^ CFU/well) under anaerobic conditions for 1 h or 3 h, respectively. Then, cell suspensions were collected and centrifuged at 10,000 rpm/min for 5 min to remove cell debris and bacteria. The release of LDH from damaged cells was measured using the LDH kit (Nanjing Jiancheng Biological Product, Nanjing, China), according to the manufacturer’s instructions. Experiments were performed in triplicate.

### 4.8. Determination of C. perfringens Adhesion to IPEC-J2 Cells

Bacterial adhesion assay was performed as previously described by Jiang et al. [104] with some modifications. Briefly, IPEC-J2 cells were seeded in 12-well cell culture plates at a density of 5 × 10^5^ cells/well. At 80% confluence, cells were preincubated with *L. plantarum* (10^7^ CFU/mL) for 6 h, after which *C. perfringens* were added into the wells (10^6^ CFU/mL) and incubated for 1 h under anaerobic conditions. Cells treated with *C. perfringens* only were used as the controls. Then, cells were washed three times using sterile PBS to remove non-adherent *C. perfringens.* Two hundred microliters of 0.25% trypsin-EDTA solution was added to the wells and digested for 15 min; then, 800 μL sterile PBS was added to each well and completely mixed. The mixed liquids were diluted for *C. perfringens* count. Each assay was performed in triplicate.

At the same time, the FITC (Solarbio, Beijing, China) labeling method was used to assay the adhesion of *C. perfringens* to IPEC-J2 cells. Briefly, *C. perfringens* culture, after centrifugation, was adjusted to 10^7^ CFU/mL with diluted FITC solution (200 μg/mL), and incubated for 2 h at 37 °C without light. Then, the bacteria were washed with sterile PBS for three times and stored at 4 °C for further use. After incubation with *L. plantarum* Lac16 for 6 h, the IPEC-J2 cells were further co-incubated with *C. perfringens* (10^6^ CFU/mL), which was labeled with FITC, for 1 h under anaerobic conditions and away from light. Then, the cells were washed three times with sterile PBS and examined under a fluorescence microscope (Nikon, Tokyo, Japan). Mean relative fluorescence intensity of FITC was determined using the ImageJ software (version 1.51, National Institute of Health, Bethesda, MD, USA). All experiments were performed in triplicate.

### 4.9. Cell Permeability to Fluorescein Sodium

Cell permeability to fluorescein sodium was assayed as previously described by Nie et al. [106] with some modifications. Briefly, IPEC-J2 cells were seeded in 12-well transwell inserts (Corning Life Science, MA, USA), with pore sizes of 0.4 mm and membrane areas of 1.12 cm^2^, at a concentration of 1 × 10^5^ cells/mL. Since IPEC-J2 cells can develop tight junctions and differentiate into tight monolayers after 9 days of culture on transwell filters [31], the culture medium in both apical and basolateral sides of the filters was renewed every 24 h for 9 days. On the 10th day, the culture medium on the apical side of the filters was removed and IPEC-J2 cells were treated as described in adhesion assay section. Then, 100 μg/mL fluorescein sodium (Sigma-Aldrich, MO, USA), dissolved in PBS, was added to the apical inserts for 1 h, after which 200 μL of medium from each basolateral side was collected. Fluorescence intensity was determined using a SpectraMax M5 (Molecular Devices, CA, USA) at an excitation wavelength of 495 nm and an emission wavelength of 525 nm. Then, we calculated apical to basolateral flux of fluorescein sodium using the standard curve. The *P*_app_ was calculated using the formula: *P*_app_ = Δ*Q*/Δ*t* × (1/*AC*_0_) [106], where Δ*Q*/Δ*t* is the permeability rate (μg/s), *A* is the diffusion area of the monolayer (cm^2^), while *C*_0_ is the initial concentration (μg/mL) of fluorescein sodium in the transwell apical inserts. All experiments were performed in triplicate.

### 4.10. Immunofluorescence Analysis

IPEC-J2 cells (5 × 10^5^ cell/mL) were seeded on glass coverslips in a 12-well flat-bottom culture plate for at least 9 days to form tight junctions. On the 10th day, the monolayer reaching polarization was treated with bacteria as described in the adhesion assay section. Cells were fixed in 4% paraformaldehyde for 20 min at room temperature and blocked with 2.5% bovine serum albumin (BSA; Solarbio, Beijing, China) for 1 h at room temperature. Cells were incubated with rabbit polyclonal anti-ZO-1 primary antibody (Invitrogen, Waltham, MA, USA) for 12 h at 4 °C, after which they were incubated with secondary antibody Alexa fluor 488 goat anti-rabbit (Abcam, Cambridge, UK) for 1 h at room temperature and away from light. Nuclei were stained with DAPI (Beyotime, Shanghai, China). Fluorescence images were obtained through laser scanning confocal microscopy (LSM 880 with AiryScan) (Zeiss, Oberkochen, Germany). Mean relative fluorescence intensity of ZO-1 was determined using the ImageJ software (version 1.51, National Institute of Health, MD, USA). All experiments were performed in triplicate.

### 4.11. PAS Staining

The IPEC-J2 cells (5 × 10^5^ cell/mL) were grown in 24-well plates (Corning Life Science, MA, USA) for 9 days and pretreated with bacteria as described in the adhesion assay section. Then, cells were washed using PBS and fixed in 70% ethanol for 10 min at room temperature. PAS staining was performed according to the manufacturer’s instructions (Beyotime, Shanghai, China). Images were obtained using a light microscope (Leica, Wetzlar, Germany). Experiments were performed in triplicate.

### 4.12. Quantitative Real-Time PCR

IPEC-J2 cells were pretreated with bacteria as described in the adhesion assay section. Then, cells were lysed using RNAiso Plus (Takara, Dalian, China) to extract RNA. Total RNA was reverse transcribed to cDNA using the HiScript II Q Select RT SuperMix (Vazyme, Nanjing, China). The qRT-PCR analysis was performed using StepOne Plus Real-Time PCR system (Applied Biosystems, USA) and the ChamQ Universal SYBR qPCR Master Mix (Vazyme, Nanjing, China). Primer sequences used in this study are shown in Table 1. All samples were run in triplicate. *β*-actin was selected as an endogenous control and relative gene expressions were analyzed using the 2^−ΔΔCt^ method [105]. Each assay was performed in triplicate.

### 4.13. Western Blot Analysis

After pretreatment with bacteria as described in the adhesion assay section, IPEC-J2 cells were lysed using the RIPA Lysis Buffer (Beyotime, Shanghai, China) involving protease inhibitors. Protein concentrations were measured using the BCA Protein Assay Kit (Beyotime, Shanghai, China). Briefly, protein samples were separated by SDS-PAGE and transferred onto polyvinylidene difluoride (PVDF) membranes (Millipore, Billerica, MA, USA). Membranes with migrated proteins were blocked with 5% dried skimmed milk for 2 h at room temperature and incubated overnight at 4 °C with the following primary antibodies: claudin-1 (Abcam, Cambridge, UK), occludin (Abcam, Cambridge, UK), ERK1/2 (Cell Signaling Technology, Beverly, MA, USA), phospho-ERK1/2 (Cell Signaling Technology, MA, USA), p38 (Cell Signaling Technology, MA, USA), phospho-p38 (Cell Signaling Technology, MA, USA), JNK (Cell Signaling Technology, MA, USA), phospho-JNK (Cell Signaling Technology, MA, USA), p65 (Cell Signaling Technology, MA, USA), phospho-p65 (Cell Signaling Technology, MA, USA), and *β*-actin (Abcam, Cambridge, UK). After being washed three times for five minutes using Tris-Buffered-Saline with Tween (TBST), membranes were incubated with horseradish peroxidase (HRP)-conjugated secondary antibodies for 1 h at room temperature. Then, protein bands were detected on an image system (Tanon, Shanghai, China) using the chemiluminescent HRP substrate kit (Millipore, MA, USA). Intensities of protein bands were determined using the ImageJ software (version 1.51, National Institute of Health, MD, USA). All experiments were performed in triplicate.

### 4.14. Statistical Analysis

Experimental data were analyzed by IBM SPSS Statistics 20 and presented as mean ± SD. Statistical significance between two groups was determined by two-tailed Student’s *t* test, while multiple comparisons were performed by one-way ANOVA. *p* ≤ 0.05 was considered significant. Graphs were drawn using the OriginPro 2018 software.

## Figures and Tables

**Table 1:** Primers used for quantitative real-time PCR.

Gene Name	Forward Sequence (5′→3′)	Reverse Sequence (5′→3′)	Accession Number
*β-actin*	CCAGGTCATCACCATCGGCAAC	CAGCACCGTGTTGGCGTAGAG	DQ845171.1
*pBD1*	TTCCTCCTCATGGTCCTGTT	AGGTGCCGATCTGTTTCATC	NM_213838.1
*pBD2*	TGTCTGCCTCCTCTCTTCC	AACAGGTCCCTTCAATCCTG	AY506573.1
*pBD3*	CCTTCTCTTTGCCTTGCTCTT	GCCACTCACAGAACAGCTACC	XM_021074698.1
*pEP2C*	ACTGCTTGTTCTCCAGAGCC	TGGCACAGATGACAAAGCCT	BK005522.1
*Mucin 2*	GGTCATGCTGGAGCTGGACAGT	TGCCTCCTCGGGGTCGTCAC	XM_021082584.1
*IL-1β*	AGAGGGACATGGAGAAGCGA	GCCCTCTGGGTATGGCTTT	NM_001302388.2
*IL-6*	ATCAGGAGACCTGCTTGATG	TGGTGGCTTTGTCTGGATTC	NM_001252429.1
*IL-8*	TCCTGCTTTCTGCAGCTCTC	GGGTGGAAAGGTGTGGAATG	NM_213867.1
*TNF-α*	CTGTAGGTTGCTCCCACCTG	CCAGTAGGGCGGTTACAGAC	NM_214022.1
*TLR-1*	GTCAGTCAGCACCGCAGTAA	CAGACAAACTGGAGGGTGGT	NM_001031775
*TLR-2*	TCACTTGTCTAACTTATCATCCTCT	TCAGCGAAGGTGTCATTATTGC	NM_213761.1
*TLR-4*	GCCATCGCTGCTAACATCATC	CTCATACTCAAAGATACACCATCG	NM_001113039.2
*NOD-1*	CTGTCGTCAACACCGATCCA	CCAGTTGGTGACGCAGCTT	AB187219.1
*NOD-2*	CCTTTTGAAGATGCTGCCTG	GATTCTCTGCCCCATCGTAG	NM_001105295.1

## Data Availability

All data generated or analyzed during this study are available from the corresponding author by request.

## Supplementary Materials

The following are available online at https://www.mdpi.com/article/10.3390/ijms222212388/s1.

## Abbreviations

*B. licheniformis*: *Bacillus licheniformis*; BSA: Bovine serum albumin; CFU: Colony forming units; CPE: *C. perfringens* enterotoxin; *C. perfringens*: *Clostridium perfringens*; DAPI: 4′,6-Diamidino-2-phenylindole dihydrochloride; DMEM/F12: Dulbecco’s modified Eagle’s F12 ham medium; ERK: extracellular signal-regulated kinase; FBS: Fetal bovine serum; FITC: Fluorescein isothiocyanate; HDPs: Host defense peptides; HRP: Horseradish peroxidase; IL: Interleukin; IPEC-J2: Intestinal porcine epithelial cell line; JNK: c-Jun NH 2-terminal kinase; *L. acidophilus*: *Lactobacillus acidophilus*; LDH: Lactate dehydrogenase; LFS: *L. plantarum* Lac16 fermentation supernatant; *L. plantarum*: *Lactobacillus plantarum*; MAPK: Mitogen-activated protein kinase; MRS: de Man–Rogosa–Sharpe; NF-κB: Nuclear factor-κB; NLRs: NOD-like receptors; NOD: Nucleotide-binding oligomerization domain; OD: Optical density; PAMPs: Pathogen-associated molecular patterns; *P*app: Apparent permeability coefficient; PAS: Periodic acid–Schiff; pBD: Porcine β-defensin; PBS: Phosphate-buffered saline; PCR: Polymerase chain reaction; *pEP2C*: Porcine epididymis protein 2 splicing variant C; PRRs: Pattern recognition receptors; PVDF: Polyvinylidene difluoride; RCM: Reinforced clostridium medium; TBST: Tris-Buffered-Saline with Tween; TLR: Toll-like receptor; TNF-α: Tumor necrosis factor-α; TSC: Tryptose sulfite cycloserine; ZO-1: Zona occludens 1.
