# Supplementary material for: Protective Effects of Lactobacillus plantarum Lac16 on Clostridium perfringens Infection-Associated Injury in IPEC-J2 Cells
Source: Int J Mol Sci. 2021 Nov 17;22(22):12388. doi: 10.3390/ijms222212388 (PMC8620398; doi:10.3390/ijms222212388)
Supplement: Supplementary file 1 [file ijms-22-12388-s001.zip › ijms-1416335-supplementary.pdf]

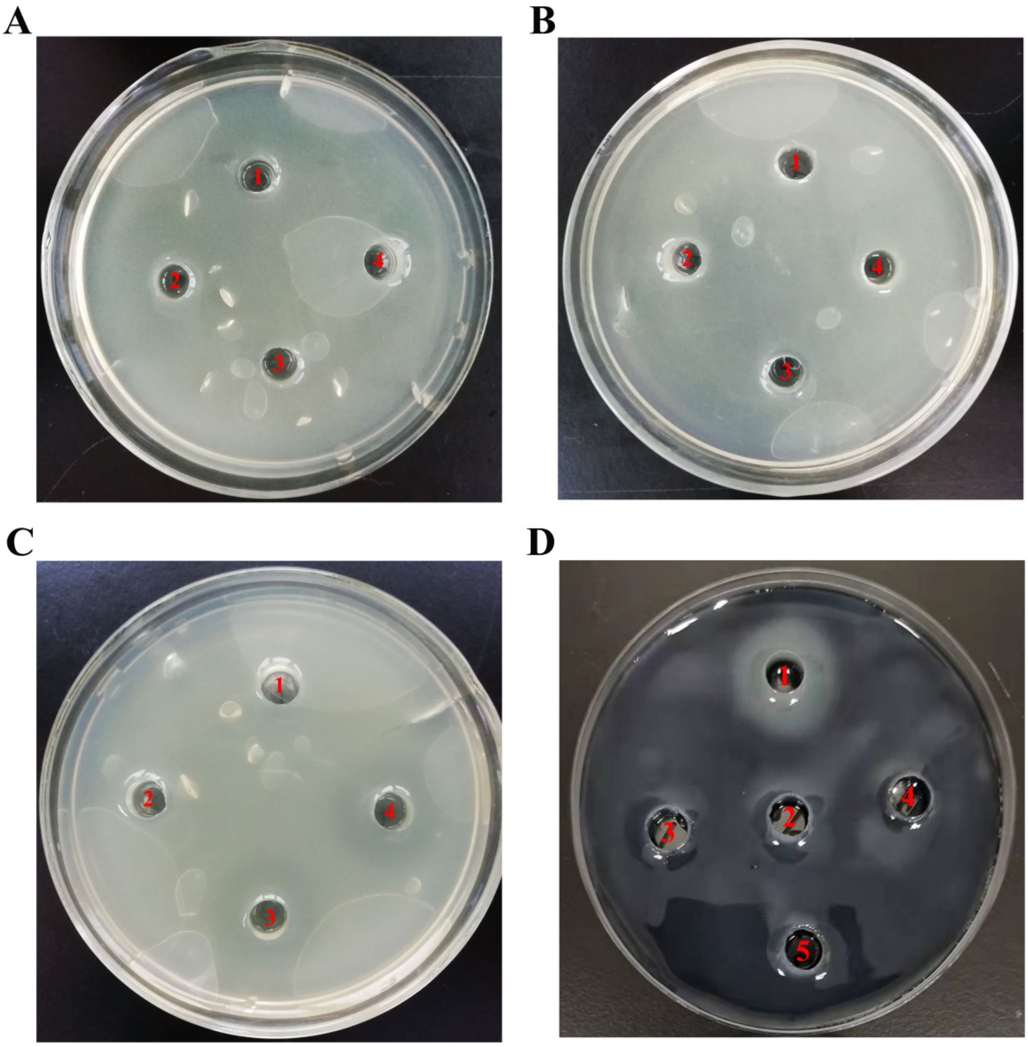

Figure S1. Agar well diffusion assays of different probiotics on *C. perfringens*. (A) *B. licheniformis*. 1 and 2: Luria-Bertani (LB) broth; 3 and 4: *B. licheniformis* fermentation supernatants. (B) *B. coagulans*. 1 and 2: LB broth; 3 and 4: *B. coagulans* fermentation supernatants. (C) *L. plantarum* Lac16. 1 and 2: MRS broth; 3 and 4: *L. plantarum* Lac16 fermentation supernatants. (D) *C. butyricum*. 1: 100 μg/mL of ampicillin; 2: RCM broth.; 3-5: *C. butyricum* fermentation supernatants.

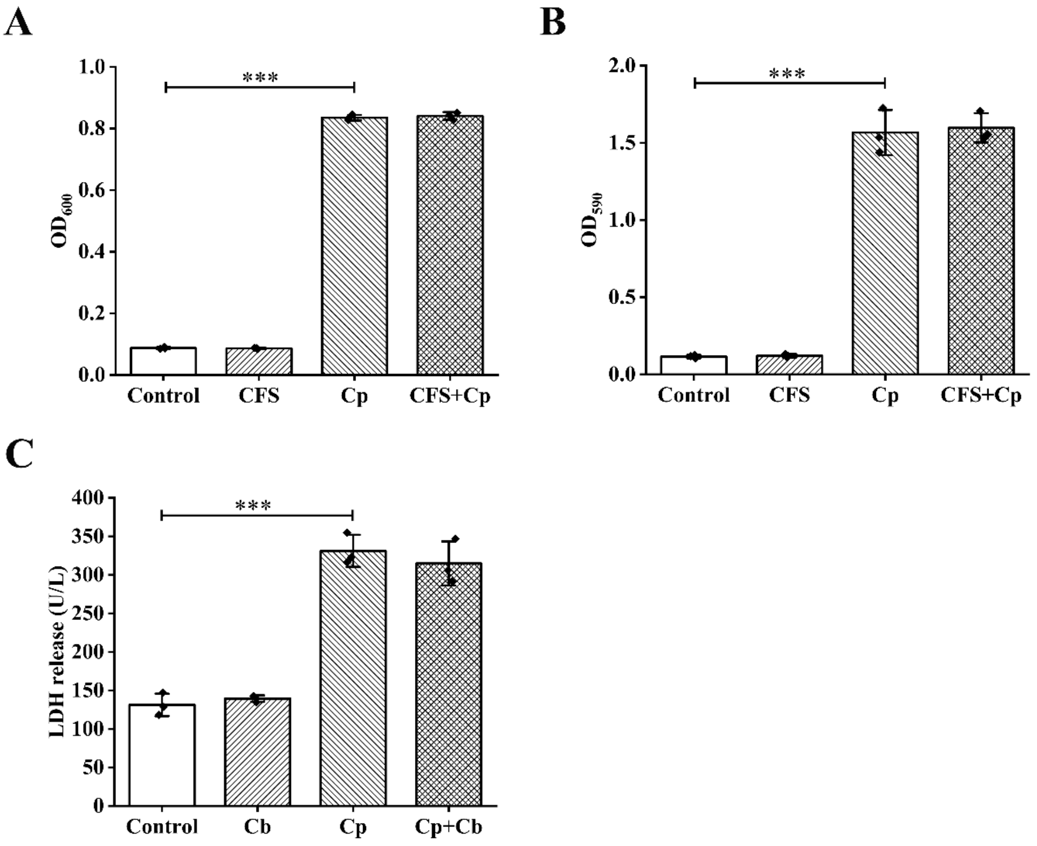

Figure S2. Evaluation of antibacterial effect of *C. butyricum*. (A) The growth of *C. perfringens* in different groups was measured by OD600 after 12 h of incubation. (B) Biofilm formation was measured by OD590. (C) Concentrations of LDH in the supernatants of IPEC-J2 cells infected with *C. perfringens* for 1 h. Data are presented as the means ± SD for n = 3; * $P < 0.05$, ** $P < 0.01$, *** $P < 0.001$ (*t* test).

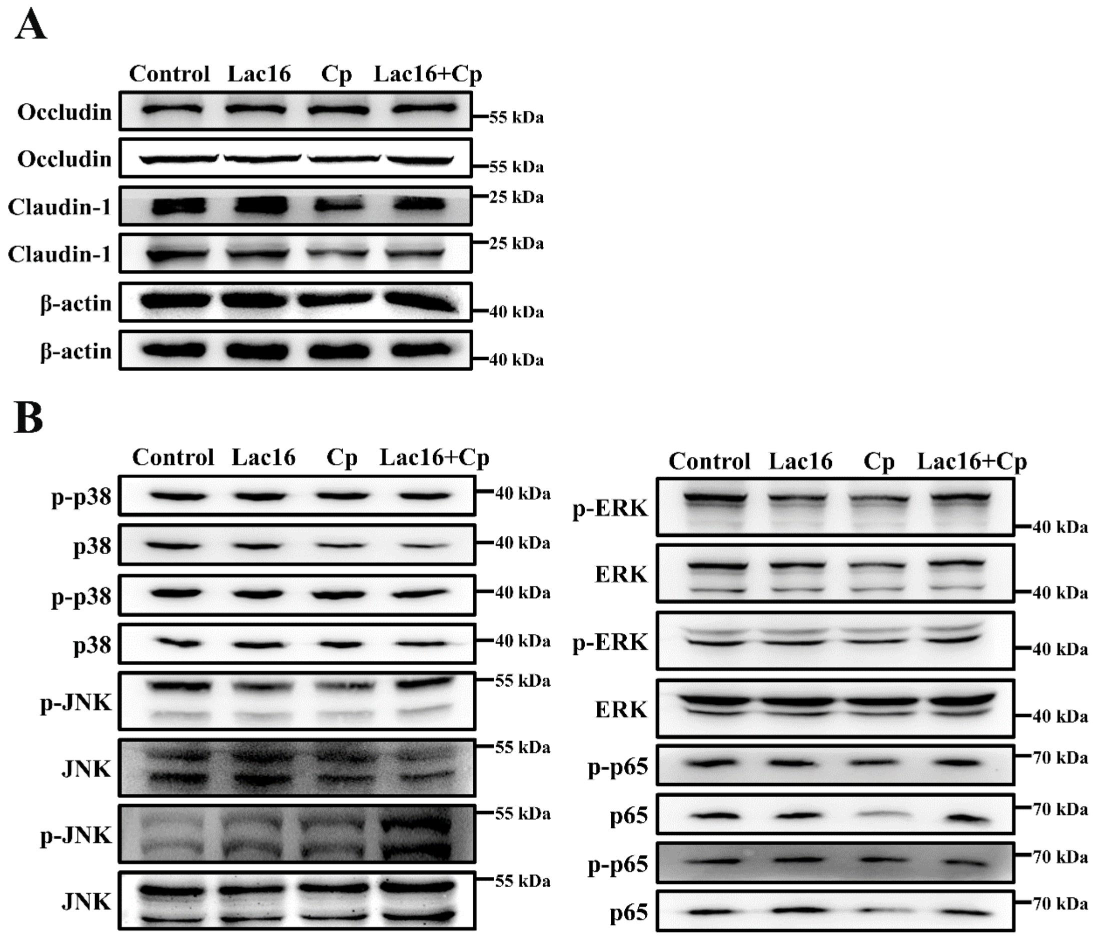

Figure S3. The rest of protein bands in the manuscript. (A) The rest of protein bands in panel D of Fig. 4. (B) The rest of protein bands in panel A of Fig. 7.
